# Indents

**Published:** 1922-06

**Authors:** 


### INDENTS

**☞Brief Articles of Technical or Scientific Value will receive notice and comment. Contributions invited.**

ORAL HYGIENE COMMITTEE OF GREATER NEW YORK.

Those members of the American Dental Manufacturers' Association who heard me address them in July, 1921, did not doubt my sincerity when I stated that I believed all Americans, no matter in what walk of life they might be found could and should seek a common ground on which to cooperate when working for the same ideals.

To do this, however, does not necessarily imply that one class is superior to another, but nevertheless each class, because of the character of its work, is better qualified to carry on particular phases of activities peculiar to its training and education.

The dental profession at no time since I have been a member of it has been ungenerous, nor has it been unmindful of the aid and services rendered to it by that body of men who represent the dental manufacturers of America.

It is therefore the more to be regretted that any manufacturer could so far forget his proper function and his real relation to the dental profession as to appear in public print claiming to be the educators of, and the beacon light for upholding and strengthening the ethics of the profession and leading it to its best standards.

Not satisfied with this alone, they have had many copies of this article printed and distributed to the members of my profession requesting them to secure its publication in their favorite newspaper.

With the seeming innocence of a child they informed me that it is unethical for me to advertise but that I may, however, derive some benefits if I secure the publication of this article in a newspaper in my town.

A western advertising firm solicits my patronage stating: "It is no longer considered unethical for a dentist to advertise, as the National Dental Association has already placed its okay on direct-by-mail advertising."

Both these firms being business houses do not understand the difference between advertising as conducted by businesses that have articles to sell, and an educational health campaign. It is for these reasons that our brothers in the trade (and the trade is an honorable profession when conducted along its own lines and for its own purposes and by its own code of ethics) should learn that it is best for all concerned, the public, the profession and the trade, if educational health campaigns be always conducted and carried on by those properly qualified by training and education to do this work, and to whom the public properly look for instruction and advice.

It is to be hoped that the majority of the Dental Manufacturing Companies of America do not agree with, nor uphold the statements made by the company claiming to be the Beacon Light of the Dental Profession, and I hope that this company will make proper and public acknowledgment of the incorrectness of these public statements.

With the kindest regards towards all men in the dental trade, and with unbounded confidence and belief in the value of public education on the importance of Oral Hygiene, I still feel that the wisest and best course to follow is to refer all public educational problems to the dental profession, and that the dental profession should and must carry on this work itself.—Thaddeus P. Hyatt, Chairman, in *Dental Digest*.

---

Business Building. Requests have often been made by dentists for ideas that may prove helpful. In this connection I would suggest the following:

First. My experience has been to the effect that a downstairs location is infinitely more advantageous than an up-stairs location. The principal advantage of the down-stairs location lies in the greater volume of patronage one thereby obtains.

Second. A system of forfeits should be established. Thus, if a patient is inclined to be antagonistic or complaining, the dentist should persevere as well as may be, and in settling charge say from twenty-five to a hundred per cent more than the usual fee. If, however, the patient is extremely acrimonious the dentist should refuse to go on with the case. Such an individual will never remain until the work is concluded, and any work that is done will never be paid for. In this regard, however, one should avoid making a mistake. When certain individuals call at a given office for the first time and their conduct is reprehensible, it may happen that after they become acquainted and confidence is established they become excellent patrons.

Third. The wearing of a large diamond, preferably on the neck scarf, has the peculiar effect of producing a large fee promptly and with but little protest. The value of a certain display in this regard has been recognized for a long time by orators, musicians, actors, salesmen and others whose vocations bring them into contact with the public. Often the individual will have an imitaton duplicate to be worn, while the genuine will be shown only on special occasions. The duplicate is often as handsome as the genuine or even more so.

These three ideas are all that I wish to present for consideration at this time. However, I would like to ruminate concerning the value of equipment and furniture. Ten years ago I inherited some money and purchased as fine equipment and furniture as was obtainable. The result was not favorable. A steady decrease in patronage followed. It seems to be maintained by certain dentists and doctors that if one desires a cheap practice that presentable furniture and equipment are a disadvantage, while a more plebian office is an advantage. This is no doubt true. There are more than a few individuals, some of them very excellent persons, who are repelled by aristocratic surroundings.

In order to break the feeling of formality, assumed culture and caste which are so repellent to many persons, I believe it is a good idea to banish all carpets and rugs from the office. Instead, we may have a linoleum or have the flfloors nicely oiled or painted. I think that if one will avoid all unnecessary display in the way of carpets, pictures and curtains and similar articles, he may have as elegent and costly equipment as is obtainable without giving offense to anyone.

In fact, I hope sometime to have an elegant and completely equipped office. It is pleasant to work in such an office and I believe that it pays.—F. C. E., in *Digest.*

---

Indirect Casting Inlays. In making inlays by the indirect method it is best to use a thimble or band slightly larger than the circumference of the tooth to confine the modeling compound. A hole should be provided at the labio-incisal or bucco-occlusal angle for the escape of the excess compound. After the impression is thoroughly chilled in place on the tooth, go over the buccal or any other surface where enamel contour has not been eliminated by your preparation, with a hot burnisher. This will soften the compound slightly where it dips into the undercuts, and the impression may then be removed without distortion of the cavity portion.

If proximal contact should be restored, take a piece of inlay wax and force it into the cavity in such a way as to get impression of the contact area of the approximating tooth. Have the patient close to give impression of occlusion, chill, trim up roughly, either just outlining, or completing the occlusal carving at this time; remove and lay aside, preferably in cold water. Now wrap a sheet of wax around your modeling compound impression in such a way as to form a tube projecting, corresponding with the root of the tooth. Build up a wall of plaster about one-half inch thick around this tube and when it is hard pack your impression with mallet force with a correct mix of good quality silver amalgam—copper amalgam may be used. When the amalgam is thoroughly hard the plaster is cut and broken away and you have your amalgam model with a projection corresponding to the root of the tooth to hold it by while forming your wax, fitting and swedging your casting, etc. Now lubricate your amalgam model with saliva or with equal parts of castor oil and glycerin. Place the wax form taken from the cavity in the mouth in place on the die. Mold it with a hot spatula into perfect adaptation around all margins; perfect your occlusal carving; go over the entire surface with a small pledget of cotton moistened slightly with Oil of Cajeput; place your spruce; cast; fit casting into amalgam model; swedge and polish upon the model.—V. C. Smedley, in *Digest.*

---

Sizing Up the Quacks. Quackery in any part of the world, but especially in America, does not die easily, notwithstanding that a continual onslaught is made upon it by reputable members of the medical profession. Respectable pharmacists, too, are mostly on the side of the doctors in regard to fraudulent nostrums, and often refuse to handle such goods even though their sale might bring them a considerable return in money.

In this connection it is interesting to note a recent article in the "Druggists' Circular," from which we quote the following:

"Members of the medical profession are like members of a fire department in that they seek to prevent the very thing which makes their calling a necessity. What would we think of a fireman who purposely permitted a conflagration in order that his comrades might show the city how important it was to keep up a well-paid fire department? And what would be thought of a man who charged the firemen of the couuntry with opposing, on the ground that it was cutting into the fireman's business, some device for putting out fires, when the firemen themselves knew the device to be a fake and an obstacle to fire prevention—a menace because of the false sense of security which it gave in time of danger?"

But the quacks do not lack confidence in themselves and their outrageous pretensions, because they know how to work upon the ignorance of the "peepul" who inhabit this land of the free and the home of the fake "cures." —*Digest.*

---

Extracting and Pregnancy. A most interesting case from the standpoint of benefit from the extraction of abscessed teeth for a woman who presented herself for examination at the seventh month of pregnancy because of dyspnea, exhaustion, and rheumatic pains in nearly every joint. General examination led to the diagnosis of mitral endocarditis with insufficiency, aortic endocarditis with insufficiency, chronic rheumatic arthritis, and marked oral and dental sepsis. The upper left gum was markedly swollen; purulent material could be expressed from many points; and most of the teeth were carious and destroyed to the gum margin. The blood pressure was 140 systolic and 75 diastolic; the pulse rate was 120 per minute. The urine showed a trace of albumin. The temperature was normal, and a blood culture was negative after forty-eight hours on a blood agar slant. The left side of the upper jaw was cleaned up at the first operation and no change of the urine, temperature, and blood culture followed. The remaining teeth and roots were later removed, sixteen in all. Improvement was marked in ten days, and she returned for delivery one month later, when examination revealed the blood pressure to be 122 systolic, 76 diastolic, and pulse rate 84 per minute. There was no dyspnea, except on exertion; the pulse was more regular, the heart was reduced in size, and compensation was restored. Two months postpartum the tonsils were removed and dental work completed for full plates.

The study of this case has led me to believe that the removal of teeth with periapical infection and extensive caries can be safely done during pregnancy. Surgical removal in such cases is preferable in the presence of definite roentgenologic evidence of granuloma. Careful roentgen-ray examination, interpretation, and co-operation with the dentist are highly essential. Surgical extractions in these cases should be done according to a definite plan, with a careful consideration of the number of teeth to be removed at one operation, the condition of the patient and her reaction following the first extraction. Repeated examination of the urine, temperature, and leukocytes should be made, and the general symptoms of reaction manifested by the patient should be carefully observed. Pyorrhea should not be overlooked and cavities in vital teeth should be filled with cement rather than with more permanent materials. In the presence of pathologic conditions associated with pregnancy, the same precautions must be taken as are observed in similar conditions existing independently of pregnancy. The eradication of foci of infection other than the teeth is equally important in the hygiene of pregnancy—W. N. Rowley, M.D., *Dental Summary*, March, 1922.

---

Annealing Clasp Gold. The spring or resiliency of the clasp is often lost when the clasp is cleaned, and when the tail or lug is soldered to it. Metal such as used for cast clasps, and having a high platinum content, is annealed by heating to a cherry red and then quickly immersing in a fifteen per cent. solution of sulphuric acid. Even sudden chilling in water will anneal it. On the other hand, it is hardened by bringing to a cherry red and then allowed to cool very slowly. So when cleaning a clasp place it in boiling acid. And after soldering to it don't throw it in acid or water, but let it cool slowly.—I. T. Dresch, in *Dental Digest.*

---

RADIUM FOR DENTAL ABSCESSES. Recent observations upon the use of radium emanation indicate that there is a possibility of employing this substance in the treatment of chronic dental abscesses, according to Dr. J. A. Marshall, associate professor of biochemistry and dental pathology at the University of California.

Experiments employing the radio-active liquid in the treatment of root canals have been conducted at the George Williams Hooper Foundation for Medical Research and at the College of Dentistry of the University of California. With one possible exception, there has been no evidence of succeeding soreness or pain—*Digest.*

---

An unclean mouth is a draw-back to health and efficiency. Yale University believes this and has accordingly decreed that every student this year must pass through the hands of the dental examiner and dental hygienist.

Many industrial plants believe it and require proper attention. Some even make a clean mouth a requisite for employment.

---

STERILIZING COTTON ATTACHED TO BROACHES. Dr. Rhein, of New York, I understand, has his secretary use sterilized rubber gloves when attaching cotton to broaches to be inserted into root canals. My method is (after the cotton is attached to the broach by the aid of the fingers) to saturate it in water and then hold over a spirit lamp flame till the water disappears in steam.

A bacteriologist, after seeing a demonstration in my rooms, expressed the view that the cotton was undoubtedly made sterile by the method shown. Thus any possibility of introducing infection into the root is eliminated.—F. A. Ormiston, in *Dental Science Journal of Australia.*

---

THE OLD WAYS. When I was young my teeth would ache as though a record they would break. Then to the village dentist's chair in trembling haste I would repair. Some simple tools were on his bench, a crowbar and a monkey wrench, a pair of pincers and an ax, a handsaw and a hunk of wax. He rolled his shirtsleeves to his neck, and gave a yank and cried, "By heck," and charged four bits and he was through—no scientific curves he knew. Now to the dentist's chair I go, to be relieved of toothache woe. He has diplomas everywhere; he has a silver-mounted chair, and instruments of every sort, and anesthetics by the quart. He talks hygiene and kindred things, and antiseptic bunk he springs; and X-ray pictures of my fangs he takes while trilling his harangues; he tries out all the little tools indorsed by modern dental schools, and fools around and chews the rag, and finally he pulls the snag. I hand him then a big fat check, and leave his joint a nervous wreck. The old-time dentist is a jest? Ah, well, I like his way the best; I'd rather have a blacksmith come and jerk a molar from its gum, than seek the dentist, in his booth, who takes a day to draw a tooth.—Walt Mason.

---

RED VULCANITE DENTURES INJURIOUS. I was interested in reading about the case mentioned in the lower part of page 764 in the December *Digest*, in which the patient has very tender lower gums. I am not sure whether this case comes within my experience, but I remember of seeing one case in the clinic at the Buffalo Dental School, in which the lady had worn upper plates of several different materials, among them gold, and she was unable to retain any of these in her mouth with comfort for any length of time. She was fitted with an aluminum plate, and reported after a time that this was more comfortable than any of the others, although she could not wear it constantly with comfort. I believe she said that she had been told that she had a "gouty diathesis." I saw no more of her after this report.

You say that "There is an occasional mouth that does not take kindly to rubber." I have seen several instances of this, the worst one of which is described on page 85 of the February *Digest*, 1921. As this patient happened to be my wife I know all about the case, and it is not overstated. She had worn red vulcanite before the plates were made, which were described on page 85, and they always gave more or less trouble, while she had none when black rubber was used. Her loss of weight during this experience, and her prompt recovery as soon as black rubber was substituted for the red showed conclusively that the trouble lay with the material used.

It is my impression that there are a great many cases of irritation from wearing red vulcanite that are not very annoying, and are consequently put up with, the patient taking them almost as a matter of course. This is simply a little piece of friendly gossip.—George B. Snow, in *Dental Digest*.

